# International Conference “Urogenital Infections and Tuberculosis” in Novosibirsk, Russia, Has Opened New Perspectives in the Fight against Tuberculosis

**DOI:** 10.3390/antibiotics3020121

**Published:** 2014-04-02

**Authors:** Ekaterina Kulchavenya, Irina Felker, Elena Brizhatyuk

**Affiliations:** 1 Novosibirsk Research Institute for Tuberculosis, Novosibirsk 630040, Russia; E-Mails: urotub@yandex.ru (I.F.); Elena.brizhatyuk@yandex.ru (E.B.); 2 Novosibirsk State Medical University, Novosibirsk 630040, Russia

**Keywords:** urogenital, infection, tuberculosis, epidemiology, diagnosis, therapy

## Abstract

The first International Conference “Urogenital Infections and Tuberculosis” was held in Novosibirsk 24–26 October 2013. Three hundred and twelve delegates from 73 cities in 16 countries took part in the conference. Actual problems of urogenital tract infection (UTI) including tuberculosis (TB) as a specific infection were discussed, including: nosocomial infections in urology, various aspects of prostate biopsy, epidemiology and diagnosis of urogenital tuberculosis, gender and age related characteristics of urinary tract infections, and male infertility, *etc*.

## 1. Introduction

The meeting of the ESIU/EAU board at the annual EAU Congress in Paris in 2013 had decided to organize the Conference on Urogenital Tuberculosis (UGTB) in a region with a high prevalence of this disease. As the Novosibirsk Research TB Institute and the TB Department of Novosibirsk Medical University are well-known for their studies in this field, and Siberia is an epidemic region of TB, Novosibirsk was chosen as a place for this Conference. The meeting was organized as a joint event between several medical associations: The European Section of Infection in Urology (ESIU)/European Association of Urology (EAU), International Commission on UTI of the International Society of Chemotherapy for Infection and Cancer (ISC), Russian Society of Urology, Russian National Association of Phthysiologists, Asian Association of Urogenital Tract Infection and Sexually Transmitted Diseases (UTI&STI), Novosibirsk Research Institute for Tuberculosis and Novosibirsk Medical University, Russia. The Organizing Committee was chaired by Professor Truls E. Bjerklund Johansen, Chair of the ESIU, Professor Kurt G. Naber, Past-President of the ISC, and Honorary Chair of the ISC WG UTI, and Professor Ekaterina Kulchavenya, as President of the Conference. The International Conference “Urogenital Infections and Tuberculosis” was held in Novosibirsk, from 24 to 26 October 2013. Three hundred and twelve delegates from 73 cities in 16 countries (Russia, Germany, Switzerland, Sweden, Israel, South Korea, China, Turkey, Ukraine, Belarus, Tajikistan and others) took part in the conference, and 191 doctors attended via on-line translation. 

One of the conference highlights included the World Health Organization (WHO) report which indicated that 8.6 million people suffer from TB in 2012, including 1.1 million cases among people with HIV. In 2012, 1.3 million people died from TB. In 2012, an estimated 450,000 people developed multidrug-resistant TB (MDR-TB) globally and there were an estimated 170,000 deaths occurred from MDR-TB.

*M. tuberculosis* may affect any organ of the human body, and next to the lungs the most common sites of the disease are the urogenital system, bones and joints. Unfortunately, because of delayed diagnosis, urogenital tuberculosis (UGTB) often has a complicated course. UGTB may mimic urogenital tract infection (UTI) with common bacteria, a fact that confuses many doctors who are not familiar with TB. 

**Figure 1:**
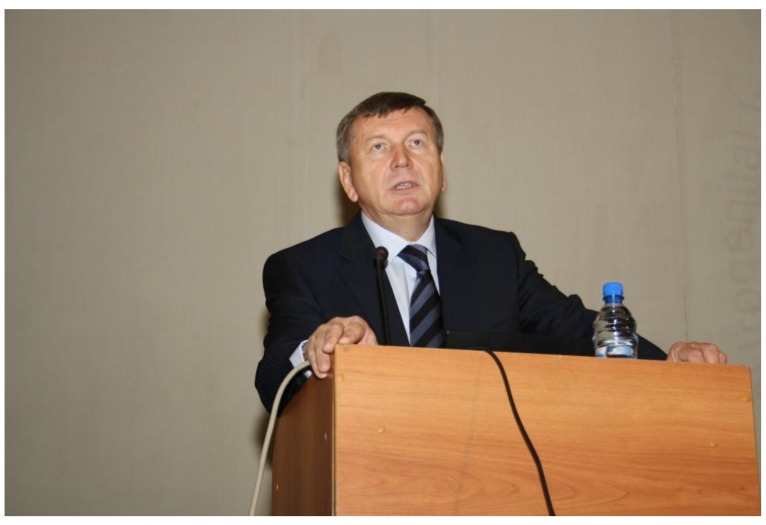
Minister of Public Health of the Novosibirsk region, Professor Leonid Shaplygin.

The official opening ceremony was attended by the Minister of Public Health of the Novosibirsk region, Professor Leonid Shaplygin (Figure 1), and the Director of the Novosibirsk TB Research Institute, Head of the TB Department in Novosibirsk State Medical University, Professor Vladimir Krasnov.

For three days there were parallel lectures, debates and discussions in two halls—in English and Russian languages with simultaneous translation. The following problems were discussed: latent urogenital infections, their bacteriological diagnosis and therapy; complicated urogenital infections, their prevention and treatment; sexually transmitted infections; infectious diseases of the reproductive system as a cause of infertility and sexual dysfunction; interrelation of infections and tumors of the urogenital system: mistakes in diagnosis and features of combined therapy; standards of examination and treatment of patients with urogenital infections; epidemiology, organization of early detection and follow up of patients with urogenital tuberculosis; classification of urogenital tuberculosis; diagnosis, therapy and surgery of urogenital tuberculosis; multi-organ tuberculosis, and multi-drug resistance (MDR) with extrapulmonary tuberculosis.

On the first day of the conference, participants were able to attend sections dedicated to the following topics: bladder leukoplakia, kidney stones, and the problem of non-specific cystitis, chronic prostatitis, and sexually transmitted infections. In the session on bladder leukoplakia, the etiology and pathogenesis of this disease were considered in addition to the role of sexually transmitted diseases. Also, the possibilities of minimally invasive procedures as well as the prospects for recovery of anatomy and physiology of the urothelium were demonstrated. A new method of less invasive intravesical laser therapy for bladder leukoplakia was presented (Ekaterina Kulchavenya, Novosibirsk, Russia) with evidence of its superiority compared to transurethral resection. Problems of urolithiasis are by no means new to urology, but they are still quite relevant. The latest techniques and the “gold standard” lithotripsy and litholytic therapy were presented and prevention of infectious complications and preoperative preparation were thoroughly discussed (Fedor Kapsargin, Igor Feofilov, Valentin Isaenko—all from Siberia, Russia). Although the topic of non-specific cystitis in urological practice has been repeatedly considered at various congresses and conferences, the contributions and discussions in this section were again very lively.

On the second day of the Congress, the followings topics were discussed: current issues of nosocomial infections in urology, various aspects of prostate biopsy, epidemiology and diagnosis of urogenital tuberculosis, gender and age related characteristics of urinary tract infections, and male infertility. Possibilities of minimally invasive and endoscopic procedures in urology, as well as modern methods of drainage of the urinary tract and wounds were demonstrated to the audience.

## 2. Highlights of the Conference

Ekaterina Kulchavenya (Novosibirsk, Russia) emphasized in her presentation on the classification of UGTB that UGTB remains an important problem, especially in developing countries, because it is often an overlooked disease. Classification includes exact description of forms and stages of the UGTB, because each stage implies a different approach of management. Thus, accurate classification is the basis for good therapeutic results. 

Kidney tuberculosis (KTB) at stage 1–2 should be treated with chemotherapy, KTB at stage 3 requires partial nephrectomy, and KTB at stage 4 is indicated for nephrectomy. A stricture of ureter needs reconstructive surgery in KTB 1–3, but nephron-ureterectomy in KTB-4. Male genital tuberculosis (MGTB) should be treated with chemotherapy; fistulas are treated by surgery. Generalized UGTB, combining both kidney TB and male genital TB should be managed depending on the forms and stages of the kidney and male genital TB. UGTB is a tremendously diverse disease. A unified standard approach is therefore impossible. The common term “UGTB” gives insufficient information about therapy needed, e.g., surgery, and prognosis—and makes evaluation of general epidemiology difficult. In contrast, a more detailed clinical classification will also improve the therapy of UGTB.

Mete Cek from Trakya University (Turkey) reported the results of a 10-year, worldwide study on the prevalence, structure and characteristics of nosocomial infections in hospitalized urological patients. Besides prevalence and contemporary classifications, also a detailed analysis was presented concerning causes of nosocomial infections, antibiotic resistance patterns of uropathogens, and international practices of antibacterial therapy.

Florian Wagenlehner from the University of Giessen (Giessen, Germany) had two presentations, one on complications of prostate biopsy and the other on new antibiotics in urology. Every year in Europe more than 1 million prostate biopsies are performed, and the risk of infectious complications after this procedure is rising. The author proposed a strategy to minimize the risk of infectious complications and justified the need of targeted antibiotic prophylaxis. In his second presentation he analyzed the global bacterial antibiotic resistance in urology in relation to the antibacterial activity of different classes of existing and new antibiotics in the pipeline. Gernot Bonkat from the University of Basle (Switzerland) presented the isothermal micocalorimetry as a new method for identification of *Mycobacterium tuberculosis* and drug susceptibility testing. According to the author’s data, isothermal microcalorimetry could displace all other culturing methods, since the minimum detection time is only 25 h and the test easily identifies the drug sensitivity of the pathogen. With regards to UGTB, with this technique the growth rate and doubling time of 4 different mycobacteria could be determined in urine simultaneously.

Topics of the third day of the conference were prevention of urogenital infections, chemotherapy and surgery of urogenital tuberculosis.

Magnus Grabe (Malmö, Sweden) discussed the prevention of infectious complications in urological surgery. He encouraged all participants to follow the recent recommendations of the EAU Guidelines.

Björn Wullt (Lund, Sweden) reviewed in his presentation the optimal management of uncomplicated UTI according to the 2013 updated EAU guidelines. Seung-Ju Lee (Suwon, Korea) described the methods of reconstructive surgery in UGTB. Denis Kholtobin (Novosibirsk, Russia) presented his rather huge own surgical experience. He emphasized the necessity of simultaneous extirpation of bladder and prostate in male patients with bladder TB.

Anna Mordyk (Omsk, Russia) paid special attention to difficulties of bacteriological confirmation of UGTB. The main reasons are: (i) rare and scant mycobacteriuria and (ii) non-optimal previous antibacterial therapy for “urogenital tract infections”, which in fact means overlooked UGTB. Olga Alhovik (Novosibirsk, Russia) presented new techniques for rapid identification of *M. tuberculosis* with BACTEC 960 and GeneXpert. According to data of the Novosibirsk Research TB Institute, in 12.5% of patients with prostate tuberculosis that initially showed negative culture results, *M. tuberculosis* was finally found with these new methods.

**Figure 2:**
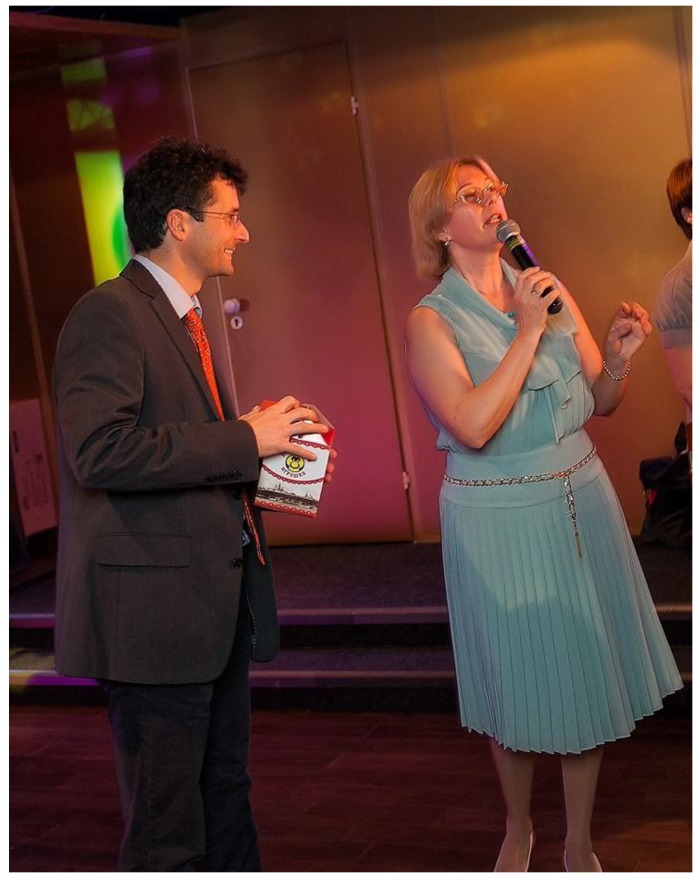
President of the Conference Professor Ekaterina Kulchavenya presents an award to Professor Florian Wagenlehner.

On Wellcome Reception after first day of the Conference some participants who made biggest contribution in this event were awarded with Diplomas and Prizes, Figure 2 shows the moment of awarding Professor Florian Wagenlehner (Germany).

## 3. Conclusions

We do not know the real incidence and prevalence of UGTB as it includes KTB, MGTB, and female genital TB. All these forms of TB have their own clinical features and require their own approaches to the diagnosis and management. Before anti-TB drugs were created, the prevalence of UGTB was huge. Every fifth urological in-patient had UGTB, more than third of all pyonephrosis were due to TB [1]. Nowadays in Siberia, about 300 patients with urological tuberculosis are revealed annually [2]. In developed countries, from 2% to 10% patients with PTB have also UGTB; in developing countries the proportion increases up to 15%–20% [3]. In Europe, UGTB is diagnosed more often in migrants, than in inhabitants [4,5]. About 20% of patients cured from PTB, had EPTB later, mostly UGTB [6]. 

This first conference brought together urologists and TB specialists not only from the Siberian region, but also from the whole of Russia and other countries as well. The outstanding quality of the presentations and the lively discussions during the congress underlined the high degree of relevance of this event. Participants were able not only to gain useful information, but also to find new partners for further joint research.

There are many un-solved problems in TB urology. Unique terminology, classification, and approach to diagnosis, therapy and surgery for patients UGTB are needed. Heated discussions, hot debates and arguments between participants at this Conference resulted in agreement on key points.

The take home messages emphasized the need for more awareness regarding UGTB in many regions of the world, considering that UGTB is a sexually transmitted disease, as *M. tuberculosis* can be found in the ejaculate of half of the TB patients [7]. A better classification of UGTB is also needed to improve treatment recommendations and report the outcome of surgical interventions, particularly for TB of the bladder and prostate. 

**Figure 3:**
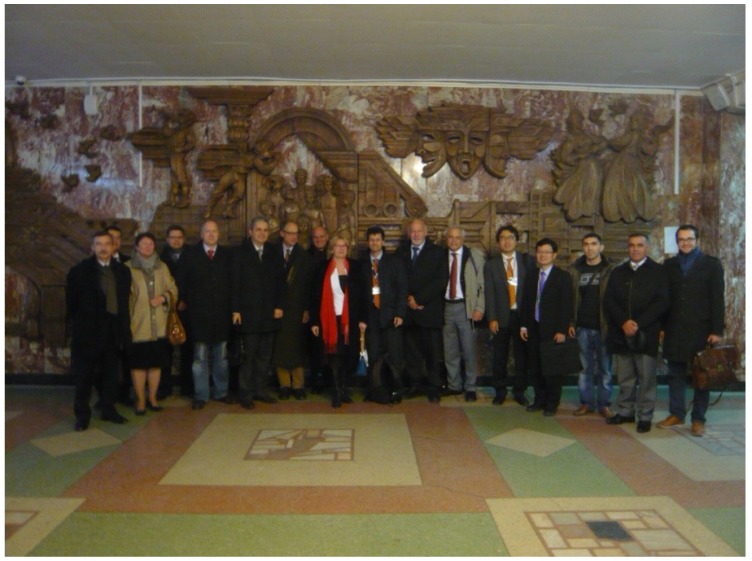
Conference faculty in the conference hall in Novosibirsk.

After Conference memory picture of the faculty was made in the hall of the Congress Center (Figure 3).

## References

[1] Marion G. (1940). Traite d’Urologie.

[2] Kulchavenya E. (2013). Best practice in the diagnosis and management of Urogenital Tuberculosis.

[3] Figueiredo A.A., Lucon A.M. (2008). Urogenital tuberculosis: Update and review of 8961 cases from the world literature. Rev. Urol..

[4] Lenk S. (2011). Genitourinary tuberculosis in Germany: Diagnosis and treatment. Der Urologe. Ausg. A.

[5] Singh D.D., Vogel M., Müller-Stöver I., El Scheich T., Winzer M., Göbels S., Hüttig F., Heinrich S., Mackenzie C., Jensen B. (2011). TB or not TB? Difficulties in the diagnosis of tuberculosis in HIV-negative immigrants to Germany. Eur. J. Med. Res..

[6] Lenk S., Schroeder J. (2001). Genitourinary tuberculosis. Curr. Opin. Urol..

[7] Aphonin A.B., Perezmanas E.O., Toporkova E.E., Khodakovsky E.P. (2006). Tuberculous infection as sexually transmitted infection. Vestn. Poslediplom. Obraz..

